# Videoautopsy—A Minimally Invasive Autopsy Method Using Endoscopic Techniques in Forensic Medicine: Clinical Features

**DOI:** 10.3390/diagnostics14090884

**Published:** 2024-04-24

**Authors:** Paweł Świderski, Szymon Rzepczyk, Beata Bożek, Czesław Żaba

**Affiliations:** Department of Forensic Medicine, Poznan University of Medical Sciences, ul. Rokietnicka 10, 60-806 Poznań, Poland

**Keywords:** autopsy, post-mortem endoscopy, minimally invasive diagnostics, post-mortem diagnostics, cause of death

## Abstract

In light of falling global autopsy rates, one of the causes of which is the resulting body disfigurement, it has become crucial to search for new, minimally invasive post-mortem diagnostic tools. One of these methods is videoautopsy, a minimally invasive autopsy technique using endoscopic methods. In the years 2020–2023, 15 videoautopsies were conducted at the Department of Forensic Medicine of the Poznan University of Medical Sciences in order to determine the usefulness of the method in forensic approaches. Each post-mortem examination included laparoscopy and thoracoscopy, followed by a classic autopsy to assess the effectiveness of the method. In total, the endoscopic examination allowed for determining the cause of death in 53.3% of cases, and when the cause of death was located in the abdominal cavity or chest, the percentage increased to 80%. Traumatic lesions had good recognition efficiency. In addition, it was also possible to collect material for histopathological and toxicological tests. Retroperitoneal organs were difficult to assess. The main limitation of the method is the inability to assess the inside of the skull and the structures of the central nervous system. Videoautopsy may become an important tool in post-mortem diagnostics and in forensic cases, especially when the alternative is to not perform an autopsy. Further research is necessary to standardise the examination protocol, optimise the instrumentation, and assess the potential synergistic effect with other methods of minimally and non-invasive post-mortem examination.

## 1. Introduction

Performing an autopsy, which is the basic and most important tool in post-mortem diagnostics, allows for precise determination of the circumstances and cause of death [1,2]. This applies both to deaths due to illness, in which post-mortem diagnostics are aimed at verifying clinical diagnoses and the correctness of the therapeutic methods used, and to situations in which there is a suspicion of criminal contribution to death [3,4]. These situations include deaths resulting from accident-related injuries and cases of homicide and suicide. Therefore, at the request of law enforcement authorities, a forensic autopsy is ordered. Its main objectives, apart from precisely determining the cause of death, also include determining whether third parties could have contributed to the death and securing evidence in order to prepare an expert opinion for the purposes of the investigation [5]. However, in recent years, the total number of autopsies performed has been decreasing [6,7]. Moreover, this phenomenon also applies to forensic autopsies [8]. According to classic autopsy methods, extensive incisions are required to visualise and examine internal organs. However, with the development of medical sciences, post-mortem diagnostic techniques are also evolving [9]. Since the end of the 20th century, minimally invasive methods have been introduced into post-mortem diagnostics [10]. This also includes minimally invasive autopsy using endoscopic techniques [11,12]. It involves inspecting the abdominal and thoracic cavities by making small incisions in specific locations to guide trocars for the optical system and instruments. This enables the assessment of the condition of the organs and the collection of samples, e.g., for toxicological tests, in a way that minimally distorts the body. However, there is currently little published research on the use of minimally invasive autopsy techniques. Moreover, some of them were published when endoscopic techniques were not as widespread as they are today. The current development in endoscopic technologies and the increase in their availability allows for continuation of this research. The aim of the study was to determine the possibility of using minimally invasive autopsy techniques using endoscopic techniques (videoautopsy) in forensic post-mortem diagnostics

## 2. Materials and Methods

### 2.1. Data Collection

The study was conducted at the Department of Forensic Medicine of the Medical University of Poznań in the years 2020–2023. The study included 15 randomly selected cases of corpses sent to the Department of Forensic Medicine for autopsy and full post-mortem diagnostics. Only paediatric cases and corpses with advanced stages of putrefaction were excluded from the study. No other exclusion criteria were used. The examinations were performed depending on the availability of staff, the mortuary, and the corpses. Moreover, performing additional diagnostics as part of the study could not delay the performance of a conventional autopsy using classical methods. All research work was approved and performed with the consent of the relevant university authorities and representatives of law enforcement agencies, including the head of the Forensic Science Department and the Prosecutor’s Office.

### 2.2. Operating Team

**Figure 1:**
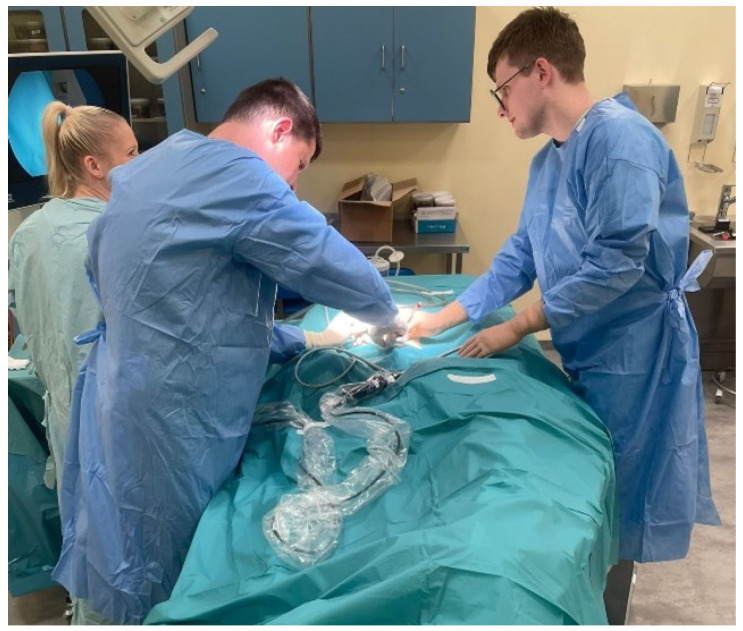
Operating team.

Each post-mortem examination using endoscopic techniques was carried out with the participation of a three-person team (Figure 1). The autopsy team was managed by the main operator, who was a forensic medicine specialist with over 15 years of experience. His tasks included making key decisions regarding the selection of instruments used during the procedure, indicating the location of trocars and methods of access to organs, managing the surgical site, and assessing the traumatic and pathological changes visible during the examination. Another member of the team was an assistant operator supporting the main operator. The function was usually performed by a forensic medicine resident or intern. His tasks included preparing corpses for examination, preparing and inserting trocars supervising the visual track and field of view, and supporting surgical activities when it was necessary to use more than two tools. The third, important member of the team was also an operating nurse specialist with years of experience in surgical wards and operating theatres. Her tasks included preparing the autopsy room and a table with surgical instruments, transferring tools during autopsy, maintaining the cleanliness of the optics, and managing the laparoscope equipment and flow of gases necessary for inflation of the corpse to facilitate the vision. Moreover, due to her education and practical experience, she participated in consultations with the chief operator on optimal trocar locations and access to organs.

### 2.3. Surgical Instruments

**Figure 2:**
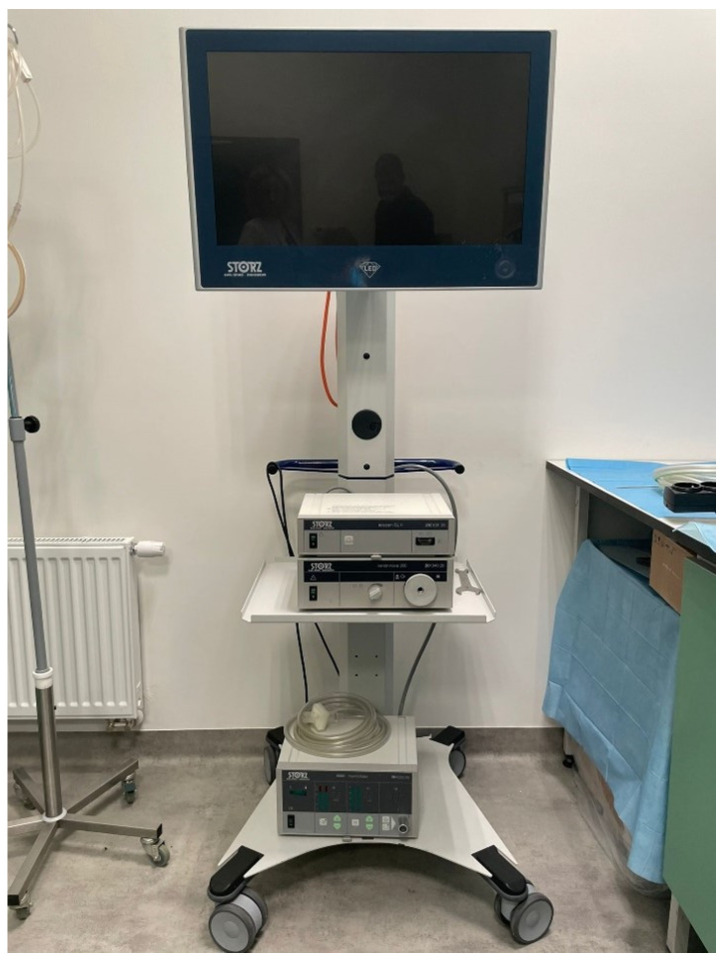
Laparoscopic column.

**Figure 3:**
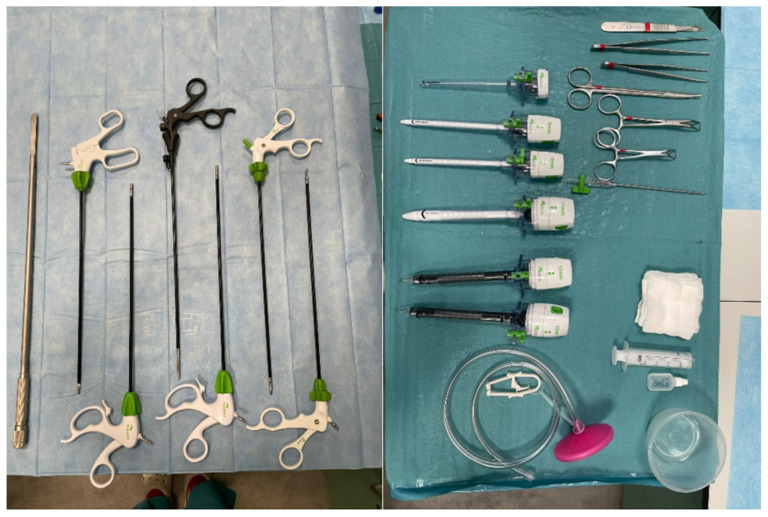
Laparoscopic instruments.

To perform post-mortem diagnostics, the STORZ brand laparoscopic column was used; the laparoscopic tower consists of a video processor (telecam SLII 202130 20), HD camera, source of light (xenon nova 300 201340 20), monitor (model 9627NB/KS-27), and CO_2_ insufflator (thermoflator 264320). The devices were placed on the mobile trolley for medical apparatus (Figure 2). Complementing the vision path, a camera head and laparoscopic optics were connected to the light source and video processor; the optics were 0 degrees (10 mm) and 30 degrees (5 mm), and the fibre-optic cable was a separate element of the set. The instruments used during the procedure were produced by Mölyncke company (Mölnlycke Health Care, Gothenburg, Sweden). The laparoscopic instrument (Figure 3) used for the study is presented in Table 1. The gas used for the inflation of the body cavities was CO_2_ due to its non-flammability, low reactivity, safe mixing with putrefactive gases, and easy access.

### 2.4. Post-Mortem Examination Protocol

Before performing the endoscopic examination, the corpse had to be properly prepared for the procedure. This included taking the body out of the morgue early enough to warm it up and placing it on the autopsy table in the position in which the examination would be performed. This was important because a too low body temperature (the temperature in the mortuary is maintained at 0 to 4 degrees Celsius) would cause significant stiffness of the integuments and subcutaneous tissue, which makes body insufflation difficult and limits the field of vision. Moreover, before the examination, the body was undressed and washed (after taking photographic documentation). Additionally, before starting the examination, the circumstances of the death were reviewed based on the analysis of materials provided by the prosecutor. Then, the laparoscopic examination was performed. The first stage was the assessment of the abdominal cavity and its organs: liver, spleen, stomach, intestines, and then the pelvic organs. Their morphology, including colour and consistency, was assessed to search for trauma and disease. Additionally, the presence of fluid and adhesions in the peritoneal cavity was evaluated. To examine the abdominal cavity, access was usually above or below the navel and on both sides of the abdominal wall in the midclavicular line. The next stage was thoracoscopy performed through the intercostal spaces or through the diaphragm. Trocars were usually located in the anterior axillary line. At that time, the condition of the lungs and pleural cavities as well as the heart with the main vessels was assessed. The assessment included a description of the condition of the organs, with particular emphasis on injuries and lesions. Additionally, during some examinations, additional tests were performed, such as cardiac puncture, laryngoscopy, and examination of the contents of the large intestine. Blood and organ samples were also collected for further tests. A conventional autopsy was then performed to compare the diagnoses. The laparoscopic examination could not delay the performance of classic diagnostics. Therefore, the minimally invasive examination was performed in the evening or early in the morning so that the period between examinations did not exceed 12 h. The comparison of the accuracy of the methods included only the analysis of the abdominal and thoracic cavities. Due to the lack of a developed endoscopic technique for assessing the inside of the skull, the assessment of injuries located in the head was not included in the analysis of clinical effectiveness.

### 2.5. Reliability Evaluation

To determine the diagnostic reliability, a three-level scale was used, proposed by colleagues from Augsburg, Germany (study conducted by Rentschler’s research group) conducting similar work on post-mortem minimally invasive diagnostics using endoscopic techniques in order to be able to compare the results between centres conducting research on the topic [13]. Grade 1 meant full agreement in detecting changes that were key to determining the cause and circumstances of death as well as incidental findings using endoscopic techniques and conventional autopsy. Grade 2 included cases where key lesions were found to determine the cause of death using minimally invasive techniques, with incidental findings remaining undiscovered until conventional autopsy. Grade 3 defined cases in which no key changes contributing to death were found during laparoscopic examination compared to traditional autopsy [13].

## 3. Results

### 3.1. Demographic Profile

A total of 15 cases were included in the study, of which 12 were male and three were female. In addition, the mean age in the study group was 39.1 years. Detailed characteristics of the corpses were included in the study, and the circumstances of death are presented in Table 2.

### 3.2. Establishing a Procedure for Minimally Invasive Post-Mortem Diagnostics Using Endoscopic Techniques

**Figure 4:**
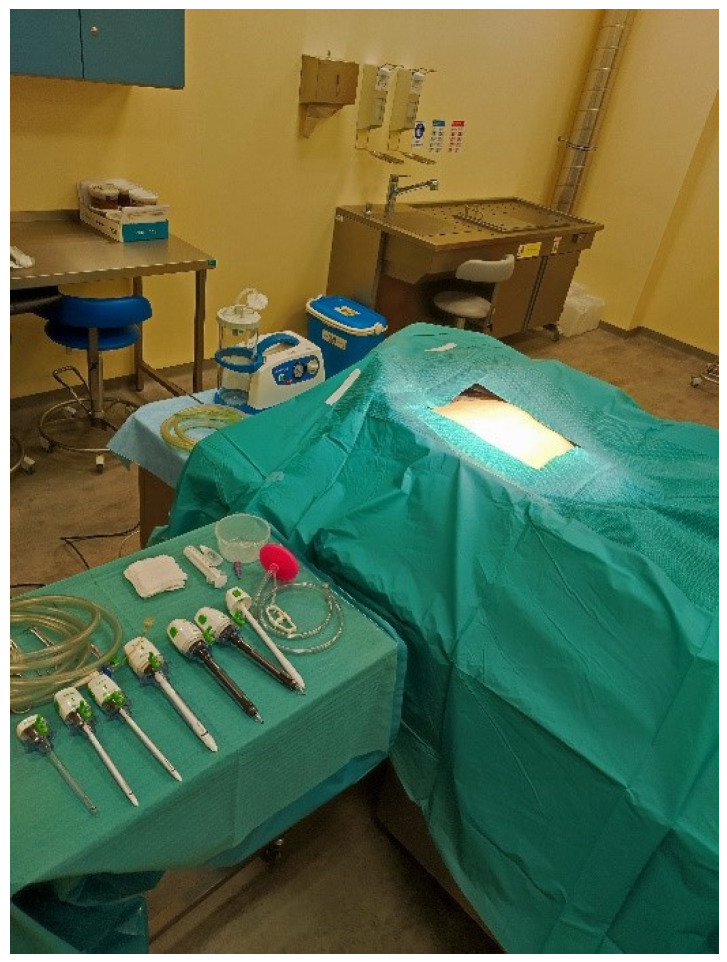
Field prepared for videoautopsy.

In each case, the body subjected to minimally invasive autopsy using endoscopic techniques was placed in the supine position (Figure 4).

**Figure 5:**
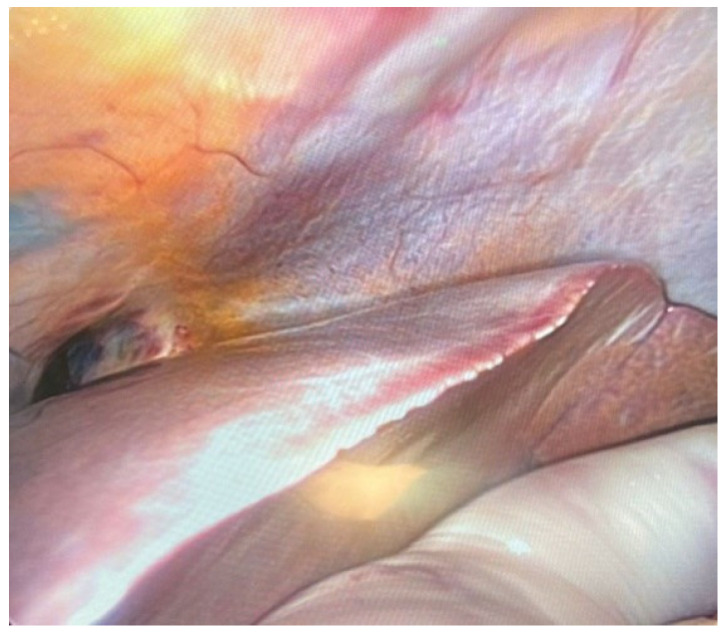
Liver assessment.

First, an abdominal assessment was performed. It was usually performed via an above-the-umbilical approach (using a Veress needle or minilaparotomy) for the largest trocar for the visual track and, depending on injuries or the presence of adhesions, two to four additional smaller trocars for instruments usually located in the lateral parts of the abdominal and upper abdominal walls. The first stage was to assess the presence of adhesions; fluid in the peritoneum; and large, significant organ injuries. The liver, gallbladder, and bile ducts were assessed successively (Figure 5).

**Figure 6:**
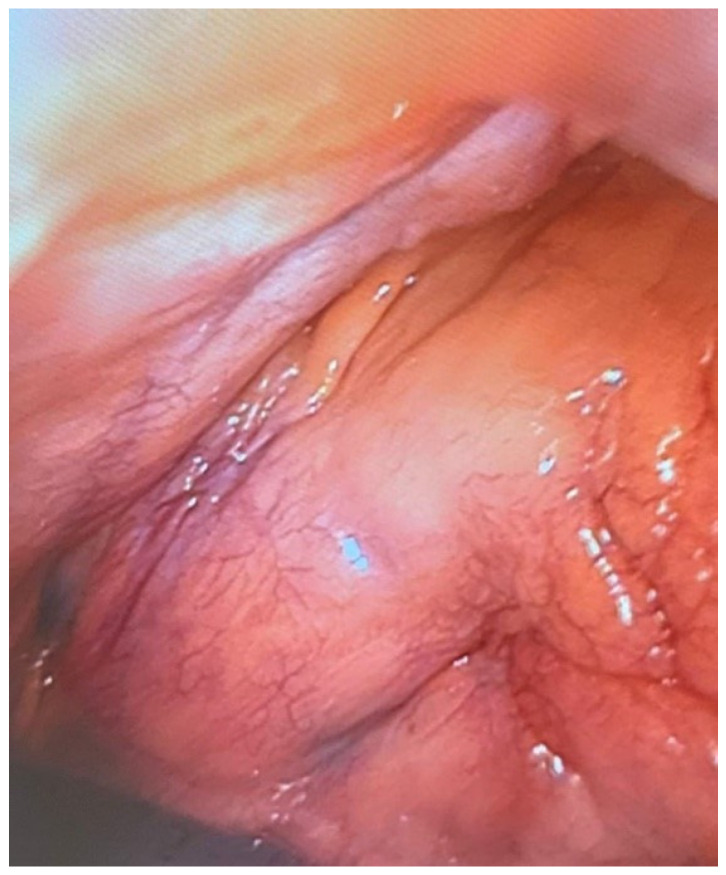
Corona cordis.

Then, the spleen was examined to assess its morphology. Attempts were also made to assess the kidneys and pancreas, which due to the location of the organs, could remain inconclusive. In order to avoid contamination of the field of view, organs filled with content, i.e., individual elements of the digestive system (stomach, duodenum, intestines), were opened as the last stage of abdominal examination. During abdominal examination, both the liver and spleen were well-imaged, especially their external surface. In both organs, traumatic changes such as lacerations were clearly visible. Additionally, in each case, it was possible to take samples for histopathological examination. The presence of fluid in the peritoneal cavity could also be detected during endoscopic examination. If adhesions were present, they were bluntly released to visualise the organs. The next stage was a chest examination. It was performed using two different methods, taking into account the degree of damage to the chest skeleton, which was crucial in cases of accidents where there was a risk of damaging the camera with bone fragments. The first method was the endoscopic approach from the diaphragmatic side. The second technique was to place the trocar in the anterior axillary line in the intercostal space. Moreover, depending on the extent of rib fractures, the decision regarding the location of trocars was made individually for the body, taking into account the best possible imaging of the interior. In the first, the mediastinum was assessed in terms of anatomical relationships and traumatic changes such as hematomas. The next stage was to assess the morphology of the lung surface in order to find, for example, emphysematous blisters or subpleural petechiae. The examination then included the lung cavity and the main bronchi. The pericardial sac was then opened to reveal its contents. The initial sections of the main vessels departing from the crown of the heart (lat. corona cordis) were well-imaged (Figure 6).

**Figure 7:**
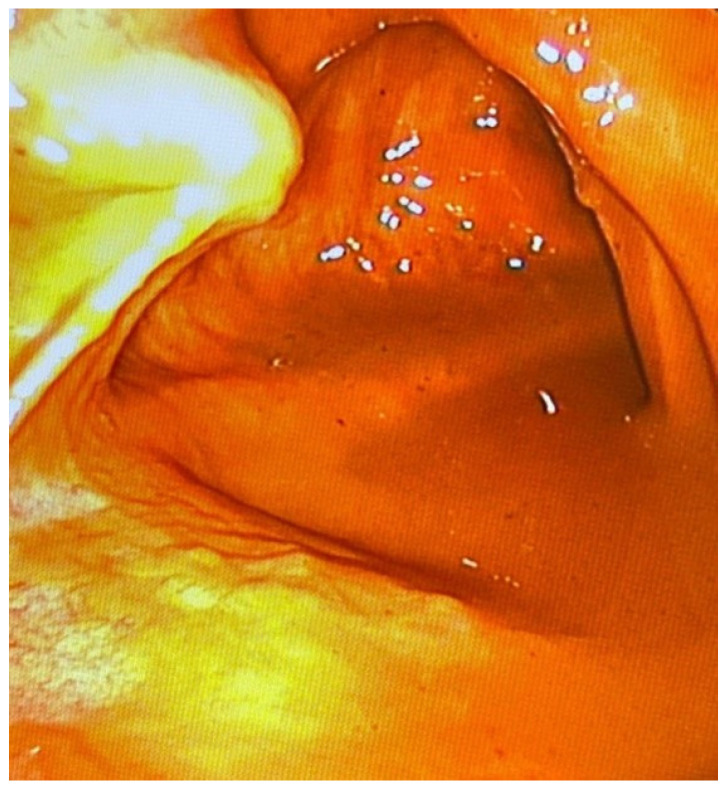
Assessment of colon content.

At this stage, the heart was assessed mainly for visible injuries, e.g., ruptures. Assessment of coronary vessels using this method has currently been omitted as a separate issue, beyond the aim of the current study, due to the high level of complexity of the examination. Additionally, during the examination of the heart, its ventricles were punctured to collect blood for laboratory tests, including toxicological analyses, which were of particular importance when the autopsy results were inconclusive. During the chest examination, traumatic lesions, e.g., rupture of the pericardial sac, wounds penetrating the heart and lungs, or foci of contusion or rupture of the lungs, were subject to good imaging. The examination also revealed subpleural lesions. Moreover, the main vessels originating in the crown of the heart, i.e., the pulmonary trunk and the ascending aorta, were well-imaged. Moreover, it was possible to collect blood from these vessels by making a simple puncture. It was also possible to assess haemorrhages accompanying broken ribs. In special cases, the minimally invasive diagnostic technique was modified in accordance with the information provided by investigators regarding the cause of death. In the case of death in a fire (case 11), laryngoscopy and a soft endoscope examination were performed to determine the presence of soot in the respiratory tract, especially in the larynx, as a feature of the vital effects of fire and death in fire. This examination confirmed the presence of soot and, therefore, death as a result of fire. In the same case, the left ventricle was punctured under the control of the visual pathway in order to collect blood for a test to determine the concentration of carbon monoxide haemoglobin. The test result showed its presence at levels exceeding lethal concentrations. In the case of suspected poisoning with oral pharmacological agents and narcotics, the contents of the gastrointestinal tract were assessed at several levels in order to detect a possible tablet mass (case 14) (Figure 7).

An endoscopic examination of the contents and wall of the large intestine was also performed by puncture with a trocar and insertion of a vision system, which allowed for a detailed assessment of the mucous membrane. The examination did not show the presence of conglomerates of a tablet or changes in the gastrointestinal tract. Moreover, it was performed as the last element of a minimally invasive post-mortem examination due to significant contamination of the visual field.

### 3.3. Clinical Efficiency

Minimally invasive post-mortem examination using endoscopic techniques allowed for determining the cause of death in eight out of 15 cases (53.3%). However, out of seven cases in which videoautopsy did not show the cause of death, the cause of death was craniocerebral injuries in five cases, which are beyond the diagnostic capabilities of the tested method. This means that in eight out of 10 cases (80%) where the cause of death was located in the abdominal cavity or chest, a minimally invasive autopsy using endoscopic techniques allowed the cause of death to be detected. In addition, in the other two cases, conventional autopsy was also inconclusive, and detailed toxicological and histopathological tests were necessary to determine the cause of death (case 5, case 14). Blood collected during minimally invasive diagnostics by coronal puncture of the heart was used for toxicological tests. The possible causes of death included extensive chest injuries, multi-organ injuries, and death in a fire. In seven cases (46.7%), minimally invasive autopsy using endoscopic techniques showed full compliance of the findings compared to classic autopsy in terms of abdominal and thoracic organs. In four cases, additional findings not related to the cause of death but found during conventional diagnostics were omitted. Only in four cases did the endoscopic examination omit significant changes that could have been associated with death. However, this mainly concerned multiple multi-organ injuries with a significant degree of damage and when the lesions were located in hard-to-reach spaces, such as the retroperitoneal space or paraspinal space in the upper part of the chest. What is more, there were cases where, despite the complete agreement of the findings of traditional and minimally invasive post-mortem diagnostics, the cause of death could not be determined without additional tests, which was the case of poisonings. Minimally invasive autopsy also allowed the cause of death to be determined, especially in cases of death resulting from trauma, despite the omission of significant additional lesions found during conventional examination.

### 3.4. Challenges

During the examination the abdominal cavity, imaging of retroperitoneal organs, i.e., the kidneys, adrenal glands, and pancreas, posed significant difficulties. Additionally, the abundant fat capsule surrounding the kidneys constituted an additional limitation, making access to the surface and cavity of the organ difficult. In the case of the pancreas, apart from the complicated access, the factor limiting access was the intestines distended with putrefactive gases. Piercing them and evacuating the accumulated gas is only possible at the end of the examination of the abdominal organs due to the possibility of contamination of the visual field with intestinal contents. Furthermore, a significant factor that complicated the examination was patient obesity. This is due to two main reasons. First of all, abundant adipose tissue requires a longer preparation time between the examination and removal of the body from the mortuary because it additionally stiffens the body in the cold, limiting the insufflation of the abdominal cavity. The second factor is the operator’s limited ability to manoeuvre the tools. When conducting abdominal examination, one should watch out for accidental perforation of the intestines. In the case of corpses, the intestines are often filled with a significant number of gases as a result of putrefaction and are more susceptible to perforation due to decomposition changes. In the event of accidental perforation, intestinal contents escape into the abdominal cavity, making it difficult to assess the organs, creating artifacts and significantly limiting the field of view, and contaminating the camera’s visual path. Furthermore, intestinal distension with putrefactive gases negatively affects the ability to image the interior of the abdominal cavity. Examination of the interior of the parenchymal organs was also a significant difficulty, especially in cases where they are also hypertrophied. During a chest examination, a critical element is to assess the heart. Currently, there is no effective and convenient technique for endoscopic heart examination. Determination of the dimensions, mass, and thickness of the heart wall along with assessment of its cross-sections is only possible during diagnostics using a traditional autopsy. This is particularly important in the case of myocardial infractions and critical narrowing of coronary arteries. Further research to develop techniques for endoscopic coronary assessment is also crucial, as the coronary vessel pathologies are responsible for a great number of sudden cardiac deaths. The thoracic section of the descending aorta is also difficult to assess due to its location. When examining the chest after trauma (e.g., as a result of a traffic accident), one should watch out for bone fragments that may damage the laparoscope camera. Therefore, it is best to precede this examination with a chest X-ray to determine the degree of bone damage and the location of fragments. Additionally, a large amount of extravasated blood requires evacuation before organ examination can be performed to facilitate the assessment. The examination of the lumen of the digestive tract and respiratory tract could be supplemented by a flexible endoscope to assess the contents in situ, e.g., in search for blood or food content. Moreover, the presence of adhesions in the peritoneal and pleural cavities limits the possibility of visualising the organ surfaces. Furthermore, a significant limitation of the minimally invasive method is the inability to assess the skull and central nervous system, which must be performed using a minimally invasive method.

## 4. Discussion

Videoautopsy, a minimally invasive method of autopsy using endoscopic techniques, may be an important tool in post-mortem diagnostics, especially in cases where the alternative is to abandon the autopsy altogether. It is a diagnostic minimum that is a compromise between giving up the traditional autopsy that disfigures the body due to extensive cuts and the need to verify or determine the cause of death. It allows for a thorough assessment of the abdominal and thoracic organs. In addition, its effectiveness in determining the cause of death has also been demonstrated in other studies [13,14]. It has been shown to be highly effective for recognising injuries related to the chest and abdominal injuries, including haemorrhages and organ injuries [15,16]. It also allows for blood to be collected for toxicological tests and organ results for histopathological assessment. In addition, the biological material collected for testing in a minimally invasive manner is of good quality, allowing for further analysis to determine the circumstances of death [17]. This makes it possible to determine the cause of death with minimal interference with the appearance and dignity of the corpses. However, the phenomenon of the postmortem redistribution of xenobiotics should always be taken into consideration during toxicological analysis of heart blood. Therefore, blood samples for toxicological analyses should also be taken from a deep vessel, e.g., femoral vessels, as reference material. The study found the effectiveness of this method in cases of death due to injuries, including traffic accidents. Importantly, not every accident or death resulting from high-energy trauma is associated with significant damage to the body’s integuments or the external appearance of the corpse. In some cases, the injuries are limited to internal organs; therefore, minimally invasive diagnostics should also be used in such cases. This technique can also be used in post-mortem diagnostics for medico-legal purposes when death was the result of an accident, suicide, or the actions of third parties [15,16,18]. Furthermore, there have also been noticeable declines in the number of forensic autopsies [8,18]. Due to the costs and time required to issue an expert opinion after an autopsy, the authorities conducting the investigation often refrain from carrying it out. Such action may result in overlooking the situations in which another person contributed to the death. Conducting rapid diagnostics with the use of endoscopic techniques is a reconnaissance activity that allows for initial confirmation or exclusion of the actions of third parties leading to death, giving guidelines for further post-mortem diagnostics and the direction of the investigation. This is particularly important in cases that seem obvious to law enforcement agencies, such as hangings, falls from heights, or drownings, which may be considered a priori by investigators as suicide or accident, leading to premature dismissal of the case [19]. Endoscopic methods are also effectively used in cases of gunshot wounds [15]. One of the important advantages of videoautopsy is the minimal impact on the appearance of the body. The trocar insertion sites are small and easy to cover after the procedure. Moreover, the use of minimally invasive post-mortem diagnostic techniques, which avoid body disfigurement and the associated dignity of human remains, contributes to improving the acceptability of the procedure and would indirectly result in halting the decline in autopsy rates [20,21,22,23]. This also applies to paediatric cases and foetuses due to improved parental acceptance [24,25,26]. Moreover, in the case of toddlers, entire organs may be extracted from the body during the laparoscopic procedure and sent for further analysis [27]. A significant limitation of the method is the inability to assess changes in the skull and central nervous system. In such cases, the examination may be supplemented with non-invasive post-mortem imaging techniques, such as post-mortem tomography [28,29]. In addition, the vitreous humour of the eye may also be collected as additional material for toxicological testing [30]. Additionally, the inside of the eyeball can be assessed endoscopically, providing important information regarding the circumstances of death [31]. In cases of functional death, e.g., as a result of cardiac dysfunction, the examination may be supplemented with the reading of cardiac implantable electronic devices [32,33]. Importantly, performing a post-mortem endoscopic examination does not affect the results of autopsy performed using the traditional technique. Assessment of the musculoskeletal system is also a significant difficulty, but in such cases, joint assessment can also be performed using minimally invasive techniques using dedicated accesses. The examination may also be complemented by the use of flexible endoscopes to assess the respiratory tract or digestive system [34]. The assessment of the middle ear can also be carried out using minimally invasive techniques, which is particularly important in cases of death of unknown cause with suspected sepsis of unknown origin, especially among children [35]. A similar procedure may also apply to the paranasal sinuses [36]. The proposed name of the procedure needs to be verified and standardised. The name all-body-cavity-scopy (ABC-scopy) proposed by the team from Germany [13] seems to be misleading because it covers only two of the three main body cavities (thorax and abdominal cavity), excluding the cranial cavity, which is the third and no less important body cavity in the practice of post-mortem diagnostics [13]. Therefore, the authors of this work suggest using the name videoautopsy. This is motivated by the key role of the video path consisting of a camera in this method of minimally invasive diagnostics. Moreover, the entire examination can be recorded in the form of a video and stored in the form of a digital file in order to maintain the complete and accurate course of the procedure, which is key evidence in investigations and in cases of attempts to undermine the reliability of the diagnostics performed [14]. Moreover, a video camera inserted into the body cavities documents changes at close range, enabling the assessment of their morphology on the recording even a long time after the examination, which is rarely possible in cases of general recording of a traditional autopsy. Consideration should also be given to the important educational and training aspect, which is an area of cooperation for surgeons and specialists in forensic medicine or pathology [14]. Surgeons can participate in and train physicians who routinely perform post-mortem diagnostics in surgical techniques using endoscopic techniques as well as taking part in the active development of a minimally invasive post-mortem diagnostic protocol using these methods. Moreover, the development of videoautopsy techniques should take advantage of surgical methods and techniques currently used in surgery. This will also be possible thanks to cooperation with surgeons. Additionally, thanks to this, they have the opportunity to practice more in controlled and safe conditions, which may result in improvement in their manual skills and their surgical technique, which will improve the quality of operations performed on patients in hospitals. This may be particularly important in the first years of education of young surgeons. In terms of combining endoscopic techniques with non-invasive imaging diagnostic methods, cooperation with radiologists and centres dealing with forensic radiology will be important. Furthermore, the combination of these methods may help develop methods of targeted diagnostics focused on pathological or traumatic changes in specific locations visible in the imaging examination. The main limitation of the study is the small number of cases analysed, but it is an initial study that confirms the possibility of using minimally invasive endoscopic techniques in post-mortem diagnostics and indicates the necessary directions for further research.

## 5. Conclusions

Carrying out post-mortem diagnostics in a minimally invasive manner using endoscopic techniques is in some cases sufficient to determine the cause of death. Good imaging primarily involves traumatic lesions and internal haemorrhages, both in the abdominal and thoracic cavities. It is important to perform organ diagnostics in the appropriate order to maintain the clarity of the field of vision and to avoid artifacts. In addition, minimally invasive methods are also used in cases of death resulting from injuries, including those resulting from a traffic accident. Depending on the circumstances of death, modifying the technique has a positive impact on its effectiveness. What is more, it is also possible to collect blood samples for toxicological or genetic tests and organ sections for histopathological tests. The limitation of the method is the inability to assess the skull and elements of the central nervous system, which must be examined using conventional techniques. Further work is necessary to develop minimally invasive techniques using the laparoscope for post-mortem diagnostics. It is necessary to develop a protocol for such diagnostics along with a unified methodology for examining organs, including those located retroperitoneally, to which access is difficult. Additionally, further research should be based on the possibility of synergistic combination with other minimally invasive and non-invasive techniques, such as post-mortem computed tomography.

## Figures and Tables

**Table 1:** Instruments used in the videoautopsy.

Instrument	Number
Bladeless dilating tip trocar 5 mm	2–4
Universal trocar cannula and optical trocar 11–12 mm	2–4
Karl Storz 10 mm 0 degree HD laparoscope	1
Karl Storz 10 mm 30 degree HD laparoscope	1
Maryland dissecting	2
Metzenbaum scissors	1
Clinch grasping forceps	2
Intestinal grasping forceps	1
Fun retractor	1
Insufflation needles VERESS	1
Karl Storz high-flow insufflation tubing	1
Laparoscopic smoke evacuation systems	1
Laparoscopic single-use suction/irrigation tubing with suction/irrigation handle	1
Plastic laparoscopic endobag	1
Laparoscopic telescopic knife	1

**Table 2:** Characteristic of cases included in the study.

	Age [Years]	Gender	Circumstances of Death	Diagnoses during the Endoscopic Examination	Autopsy Diagnosis	Cause of Death	Grade	Was Laparoscopy Sufficient to Determine the Cause of Death?
1.	29	M	Circulatory arrest due to brady asystole mechanism associated with cranio-cerebral injuries	Distended intestines and stomach with gas, scant haemorrhage in the parietal pleura, rib fractures on the right and left side, signs of hepatic steatosis, abdominal organs without traumatic or disease changes, indicators of coal pneumoconiosis, liquid blood in the vessels	Fracture of the ribs on the right and left side, hepatic steatosis, passive congestion of internal organs, liquid blood content in the vessels, no traumatic or disease changes in the abdominal organs, pneumoconiosis	Craniocerebral injuries	I	No
2.	48	F	Sudden death due to spontaneous causes—bilateral massive purulent pneumonia	Stomach distended with gas, numerous small lung abscesses, signs of coal pneumoconiosis, focal thickening of the pleura, hepatic steatosis, bloody lines in the parietal pleura on the left side, pleural adhesions, liquid blood in the vessels	Mucopurulent contents in the lower respiratory tract, massive bilateral pleural adhesions, signs of massive bilateral purulent pneumonia with the presence of numerous abscesses and cavities in the lung parenchyma, hepatic steatosis, uterine fibroids, rib fracture on the left side and sternal body fracture, liquid blood content in blood vessels and heart chambers	Bilateral massive purulent pneumonia	II	Yes
3.	44	M	Road accident—car driver	Rib fractures, pulmonary contusions, pericardial laceration, rupture of the heart and main vessels, mediastinal hematoma, liquid in the abdominal cavity, peritoneal adhesions, subcapsular haemorrhages on the diaphragmatic surface of the liver	Hematoma of the soft tissues of the anterior mediastinum, massive hematoma of the left and right pleural cavities, foci of contusion of the right and left lung, rupture of the pericardial sac, rupture of the wall of the right ventricle, subcapsular haemorrhage of the liver, fracture of ribs I–VIII on the right and I–VII on the left side, fracture of the sternum, liquid blood in the vessels and heart chambers, gallstones, pancreatic steatosis	Chest injuries	II	Yes
4.	25	M	Road accident—car passenger	Laceration of the liver—right lobe on the diaphragmatic and visceral surface, rupture of the spleen in the hilum area, bleeding in the peritoneum, a small amount of liquid blood in the peritoneal cavity, rib fractures, lung contusion with injuries to the parenchyma, rupture of the pericardial sac with injury to the heart and main vessels	Numerous injuries to the right and left lung; rupture of the right lung hilum; scanty hematoma of the right and left pleural cavities; contusion of the left lung; rupture of the pulmonary trunk and pulmonary arteries; rupture of the pericardial sac; rupture of the walls of the right ventricle, left atrium, and left ventricle; rupture of the spleen; numerous ruptures of the liver; scanty hematoma of the peritoneal cavity; rupture of the right kidney; fracture of the sternum and ribs I–X on the right and left; content of liquid blood in the vascular bed and heart chambers	Multi-organ injuries	II	Yes
5.	22	M	Sudden death at home	No traumatic or disease lesions of the organs of the thoracic cavity apart from subpleural haemorrhages, no traumatic or disease lesions of the abdominal cavity organs, liquid blood in the vessels	Subpleural petechiae, subepicardial petechiae, passive congestion of internal organs, liquid blood content in blood vessels and heart chambers	Acute circulatory and respiratory failure—sudden cardiac death (SCD)	I	No
6.	31	M	Road accident—pedestrian hit by a car	Lung contusion, haemothorax and mediastinum, rib fractures, no traumatic or disease changes in the abdominal organs, liquid blood in the vascular bed	Haemorrhage of the soft tissues of the anterior mediastinum, massive haemothorax, contusion of the right lung, rupture of the thoracic aorta, fracture of the ribs on the left side, content of food in the respiratory tract, content of liquid blood in the vascular bed	Chest injuries	III	Yes
7.	57	M	Road accident—motorcyclist	Rib fractures, lung rupture, haemothorax, detachment of the main bronchi, damage to the pericardial sac and heart, small amounts of blood content between the intestines and in the Douglas cavity, liver damage, bleeding in the parietal peritoneum on both sides of the abdomen, bilateral rib fractures	Rupture of the tracheal bifurcation, disruption of the main right bronchus, rupture of the right and left pleura, numerous injuries to the right lung on the posterior surface, pericardial sac torn on the anterior surface, rupture of the anterior heart wall, rupture of the left atrium, rupture of the aortic arch, rupture of the right coronary artery, peritoneal cavity with a small amount of liquid blood, tearing of the right lobe of the liver, numerous superficial subcapsular tears in the parenchyma of the right lobe of the liver, multiple ruptures of the left and right kidneys, fracture of the sternum, bilateral rib fractures	Multi-organ injuries	III	Yes
8.	86	F	Road accident—car passenger (death in hospital)	Blood in the pleural cavities, mediastinal haematoma, bleeding in the area of the lung hilum, rib fractures, ruptures of the lungs, damage to the pericardial sac, bleeding in the parietal peritoneum, bleeding in the anterior abdominal wall	Massive mediastinal haematoma, bleeding in the intercostal muscles, bilateral haemothorax, rupture of the lungs, rupture of the pericardial sac, sternum fracture, rib fracture, contusion of the retroperitoneal space, liquid blood content in the vascular bed and heart cavities, atherosclerosis of coronary arteries and aorta, right kidney cyst, uterine fibroids	Chest injuries	II	Yes
9.	31	M	Fall on a hard surface—death in hospital	Blood-coloured liquid in the pleural cavities, rupture of the liver on the diaphragmatic surface of the left lobe, congestion of organs, liquid blood in the vessels	Blood-coloured liquid content in the pleural cavities, a small rupture of the left lobe of the liver, passive congestion of internal organs, content of liquid blood in the vascular bed	Craniocerebral injuries	I	No
10.	31	M	Road accident—motorcyclist	Liquid blood content and blood clots in the chest, tearing and numerous ruptures of the liver in the right and left lobes, rupture of the spleen, content of liquid blood and clots in the peritoneal cavity, intestines and stomach distended with gases, obesity	Contusion of the diaphragm, liquid blood content and clots in the pleural tract, contusion of the left lung, damage to the abdominal muscles on the left side, liquid blood content and clots in the peritoneal tract, multiple ruptures of the spleen, rupture of the right lobe of the liver with rupture of the bile ducts, ruptures of the right and left lobes of the liver, rupture of the pancreas, rupture of the left kidney, rupture of the right kidney, contusion of the urinary bladder, rupture of the mesentery of the small intestine, contusion of the cecum and ascending colon, fracture of the sternum and ribs, atherosclerosis of the coronary arteries and aorta	Multi-organ injuries	III	Yes
11.	46	M	Death in a fire	Pulmonary emphysema, passive congestion of internal organs, liquid blood content in blood vessels, abdominal organs without any traumatic or disease changes Laryngoscopy—a significant amount of black mucous content with an admixture of soot was found in the larynx. Under the control of the visual track in the abdominal cavity, the left ventricle was punctured percutaneously, and blood was collected for testing for carboxyhaemoglobin content.	Soot in the upper and lower respiratory tract, emphysema, passive congestion of internal organs, liquid blood content in blood vessels and heart chambers	Acute carbon monoxide poisoning (with concomitant acute ethyl alcohol poisoning)	I	Yes
12.	41	M	Road accident—car passenger	Blood in the pleural cavities, subpleural emphysematous bullae, abdominal cavity without visible traumatic changes, liquid blood in the vessels	Liquid blood in the left pleural cavity, rupture of the descending aorta wall, sternum fracture, rib fracture, liquid blood in the vascular bed and heart chambers	Craniocerebral injuries	III	No
13.	34	M	Road accident—motorcyclist	Lung surface and pleural cavities without significant changes, percutaneous endoscopic gastrostomy (PEG), liquid content in the peritoneal cavity, a healed surgical wound in the left mid-abdomen area, a ventriculoperitoneal valve implanted nearby	The pleural cavity free of liquid and adhesions, the abdominal organs free from any injuries or diseases, the condition after a percutaneous endoscopic gastrostomy (PEG) and the implantation of the ventriculoperitoneal shunt	Craniocerebral injuries	I	No
14.	25	F	Corpse discovered in the apartment	No traumatic changes in the abdominal cavity, large amount of gas in the stomach, condition after removal of the appendix, adhesions in the navel. Chest—lungs and pleura unchanged. Additionally, the contents of the large intestine were inspected, revealing the digestive contents.	Pleural cavity free of liquid and adhesions, lungs unchanged, condition after appendectomy, abdominal organs without traumatic or disease changes	Poisoning with the antidepressant drug amitriptyline	I	No
15.	37	M	Road accident—pedestrian hit by a car	No traumatic or pathological changes in the abdominal cavity, small amount of liquid in the pleural cavities, subpleural petechiae	No traumatic or pathological changes in the abdominal cavity, small amount of liquid in the pleural cavities, subpleural petechiae	Craniocerebral injuries	I	No

## Data Availability

The data supporting the reported results can be found in the archive of the Department of Forensic Medicine of Poznan University of Medical Sciences.
